# Prognostic value of PD –L1 expression in patients with primary solid tumors

**DOI:** 10.18632/oncotarget.23580

**Published:** 2017-12-22

**Authors:** Xiao Xiang, Peng-Cheng Yu, Di Long, Xiao-Li Liao, Sen Zhang, Xue-Mei You, Jian-Hong Zhong, Le-Qun Li

**Affiliations:** ^1^ Department of Hepatobiliary Surgery, Affiliated Tumor Hospital of Guangxi Medical University, Nanning 530021, China; ^2^ Department of Colorectal Anal Surgery, The First Affiliated Hospital of Guangxi Medical University, Nanning 530021, China

**Keywords:** primary solid tumors, programmed death ligand 1, overall survival, meta-analysis

## Abstract

Programmed death-ligand 1 (PD-L1) is thought to play a critical role in immune escape by cancer, but whether PD-L1 expression can influence prognosis of patients with solid tumors is controversial. Therefore, we meta-analyzed available data on whether PD-L1 expression correlates with overall survival (OS) in such patients. PubMed, EMBASE and other databases were systematically searched for cohort or case-control studies examining the possible correlation between PD-L1 expression and OS of patients with solid tumors. OS was compared between patients positive or negative for PD-L1 expression using scatter plots, and subgroup analyses were performed based on tumor type and patient characteristics. Data from 59 studies involving 20,004 patients with solid tumors were meta-analyzed. The median percentage of tumors positive for PD-L1 was 30.1%. OS was significantly lower in PD-L1-positive patients than in PD-L1-negative patients at 1 year (P = 0.039), 3 years (P < 0.001) and 5 years (P < 0.001). The risk ratios of OS (and associated 95% confidence intervals) were 2.02 (1.56-2.60) at 1 year, 1.57 (1.34-1.83) at 3 years and 1.43 (1.24-1.64) at 5 years. Similar results were obtained in subgroup analyses based on patient ethnicity or tumor type. The available evidence suggests that PD-L1 expression negatively affects the prognosis of patients with solid tumors. PD-L1 might serve as an efficient prognostic indicator in solid tumor and may represent the important new therapeutic target.

## INTRODUCTION

Immune co-stimulatory and co-inhibitory receptors determined the functional outcome of T cell receptor (TCR) signaling and immune surveillance [1]. Tumors can modulate the interactions between inhibitory receptors and ligands to scape immune responses [2, 3]. For example, the co-inhibitory receptor programmed cell death 1 (PD-1) plays a key role in cancer immune, especially in the immune escape phase [4]. PD-1 can be expressed in activated CD4 + and CD8 + T cells, but also in some natural killer cells and B cells [5]. When PD-1 binds to the ligand PD-L1 (B7-H1) expressed on the surface of tumors, it strongly inhibits the production of T cells and cytokines [6, 7], promoting tumor cell growth and immune escape [8, 9].

PD-L1 also plays a key role in binding to PD-1 receptors expressed on activated T cells in T cell co-suppression and depletion [9–11]. PD-L1 expressed on tumor cells promotes tumor cell-specific T cell inactivation or apoptosis, leading to tumor cell growth and exacerbation of tumor immune escape [12]. PD-L1 is expressed in many types of human cancers, including in esophageal, gastrointestinal, pancreatic, breast, lung and kidney cancers [10–14]. Clinical trials suggest that blocking the PD-1/PD-L1 interaction using anti-PD-1 antibodies can be effective against several different malignancies, including melanoma, lung cancer, kidney cancer and bladder cancer [15–19].

In addition to serving as a therapeutic target, PD-L1 may also be useful as a prognostic biomarker [22]. However, whether PD-L1 expression is associated with worse prognosis for patients with primary solid tumors remains controversial [20–22]. Therefore we meta-analyzed all available evidence to address this question comprehensively.

## RESULTS

**Figure 1:**
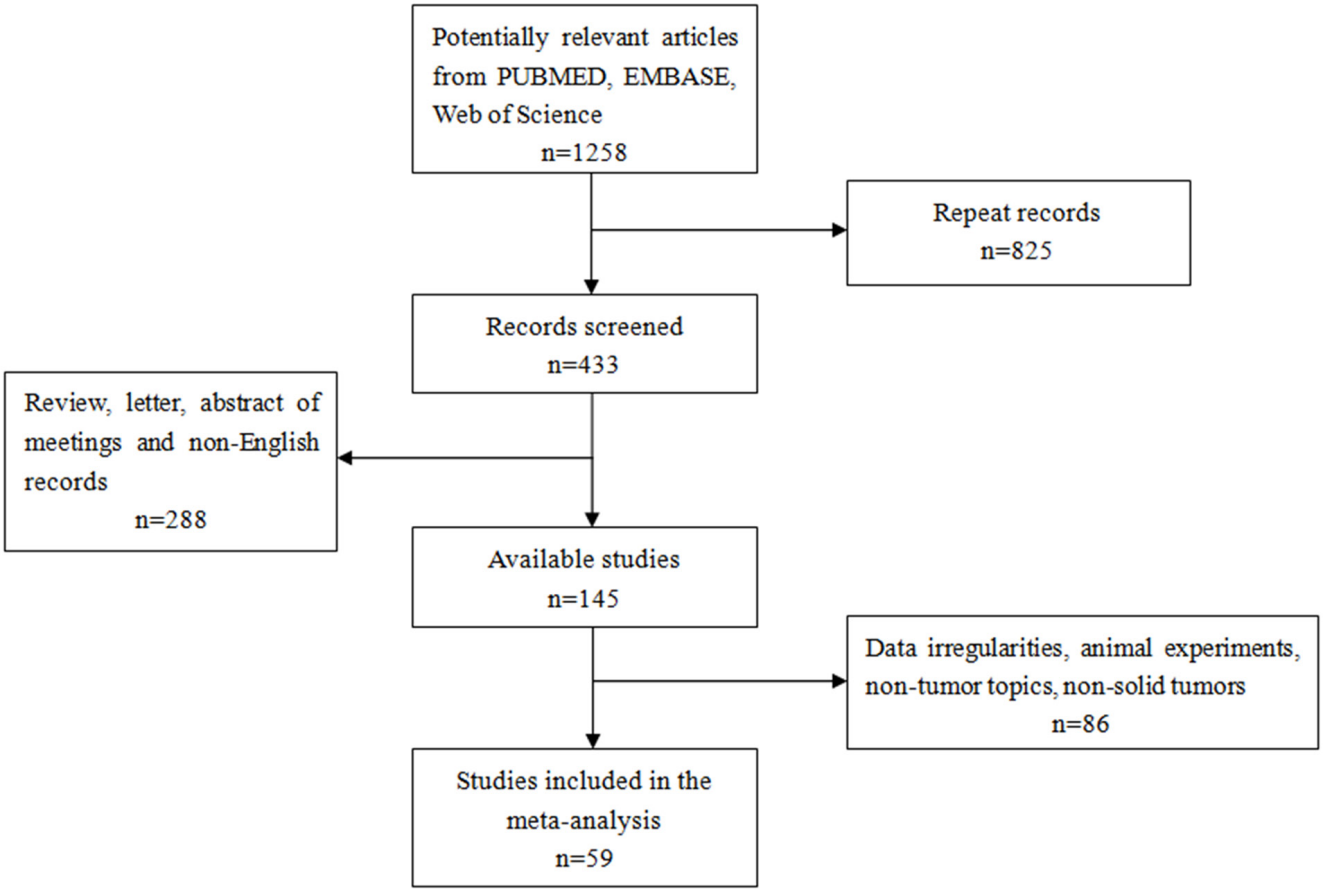
Flow chart of study selection

A total of 1,258 records were retrieved from PUBMED, EMBASE, Web of Science and EBSCO (Figure 1). After excluding 825 duplicate publications, we reviewed the abstracts and titles of the remaining 433 articles. This led to the exclusion of another 288 records that were not original research articles published in English. The remaining articles were read in full, leading to the exclusion of 86 records because they did not deal with human patients or solid tumors, or because they failed to report adequate outcomes data. In the end, 59 articles were included in the meta-analysis.

Key features of the 59 studies are summarized in Table 1; 35 studies involved Asian populations and 24 involved non-Asian populations. The studies analyzed 20,004 patients from China [23–41], France [42], New Zealand [43, 44], Brazil [45], Australia [46], Canada [47, 48], Italy [49], Germany [50, 51], United States [52–65], Japan [66–74], South Korea [75–78], Switzerland [79] and Taiwan [80, 81]. PD-L1 expression, which was analyzed in similar ways across all studies, was characterized as positive in 6,028 patients and negative in the remaining 13,976. One third of the studies (19) involved gastrointestinal tumors, while the remaining 40 involved other types of tumors. Altogether 11 malignancies were represented in the patient population: breast cancer (5 studies), renal cell carcinoma (7), colorectal cancer (3), esophageal cancer (3), gastric cancer (7), hepatocellular carcinoma (7), Merkel cell carcinoma (3), small cell lung cancer (11), oral squamous cell carcinoma (5), pancreatic cancer (3), and urinary tract epithelial cell carcinoma (4).

**Table 1:** Characteristics of studies included in the meta-analysis

Study	Country	Tumor type	Characteristic	Age	Gender male / female	No. patients positive/ negative for PD-L1	PD-L1-positive OS (%)			PD-L1-negative OS (%)			*P*
							1-yr	3-yr	5-yr	1-yr	3-yr	5-yr	
**Qin 2015**	China	Breast cancer	Primary	47(21-84)	-	189/681	100	85	81	100	98	92	<0.001
**Sabatier 2015**	France	Breast cancer	Primary	≤50: 1288 1021 (28%) 267 (31%) >50: 3207	-	1076/4378	97	90	82	97	90	81	0.070
**Muenst 2014**	Switzerland	Breast cancer	Primary	63.8 ± 14.2	-	152/498	90	55	37	98	85	80	<0.001
**Baptista 2016**	Brazil	Breast cancer	Primary	≤50: 176 1021 (28%) 267 (31%) >50: 204		107/82	98	90	85	100	96	93	0.030
**Beckers 2016**	Australia	Breast cancer	Primary	-	-	123/38	96	92	81	96	73	65	0.035
**Droeser 2013**	Italy	Colorectal cancer	Primary	69.9 (30–96)	741/673	669/1420	84	71	61	72	48	37	<0.001
**Shi SJ 2013**	China	Colorectal cancer	Primary	59.8 ± 12.5	91/116	64/143	75	54	42	90	72	61	0.017
**Zhu 2014**	China	Colorectal cancer	Primary	≤50: 54 1021 (28%) 267 (31%) >50: 47	53/48	55/46	-	-	62	-	-	80	0.051
**Krambeck 2007**	USA	Renal cell carcinoma	Primary	≤65: 54 1021 (28%) 267 (31%) >65: 47	150/148	70/228	78	62	48	91	83	76	<0.005
**Thompson 2005**	Canada	Renal cell carcinoma	Primary	-	-	103/196	84	67	52	93	87	84	<0.001
**Thompson 2007**	Canada	Renal cell carcinoma	Primary	≤65: 138 1021 (28%) 267 (31%) >65: 129	177/90	142/267	88	68	-	94	85	-	0.004
**Abbas 2016**	Germany	Renal cell carcinoma	Primary	63 (31–88)	116/61	37/140	85	57	47	92	75	66	0.005
**Choueiri 2014**	USA	Renal cell carcinoma	Primary	59 (24–81)	55/46	11/90	72	48	48	98	95	85	<0.001
**Thompson 2004**	USA	Renal cell carcinoma	Primary	-	-	87/109	87	62	-	95	92	-	<0.001
**Thompson 2006**	USA	Renal cell carcinoma	Primary	-	-	73/233	78	51	42	95	90	83	<0.001
**Ohigashi 2005**	Japan	Esophageal cancer	Primary	≤65: 24 1021 (28%) 267 (31%) >65: 17	32/9	18/41	60	18	18	88	53	45	0.001
**Tanaka 2016**	Japan	Esophageal cancer	Primary	62.6 ± 10.0	157/33	53/127	61	30	25	79	56	51	0.001
**Chen 2014**	China	Esophageal cancer	Primary	≤65: 51 1021 (28%) 267 (31%) >65: 48	76/23	79/20	100	44	17	83	44	37	0.675
**Loos 2011**	Germany	Esophageal cancer	Primary	-	-	37/64	79	51	32	96	82	69	<0.001
**Shohei 2016**	Japan	Gastric carcinoma	Primary	67 ± 14	75/30	28/105	84	41	10	91	63	51	0.022
**Geng 2015**	China	Gastric carcinoma	Primary	≤65: 65 1021 (28%) 267 (31%) >65: 35	61/39	65/100	72	41	29	87	61	37	0.026
**Hou 2014**	China	Gastric carcinoma	Primary	≤58: 55 1021 (28%) 267 (31%) >58: 56	75/36	70/111	78	46	32	93	77	68	<0.001
**Wu 2006**	Sweden	Gastric carcinoma	Primary	≤65: 64 1021 (28%) 267 (31%) >65: 38	75/27	43/102	75	38	30	98	71	64	0.001
**Tamura 2015**	Japan	Gastric carcinoma	Primary	66.1 (17-89)	305/126	128/303	90	65	49	94	78	64	0.001
**Zheng 2014**	China	Gastric carcinoma	Primary	≤60: 42 1021 (28%) 267 (31%) >60: 38	62/18	33/47	86	65	52	91	69	53	0.636
**Qing 2015**	USA	Gastric carcinoma	Primary	≤60: 42 1021 (28%) 267 (31%) >60: 38	72/35	54/107	81	28	18	93	47	27	0.004
**Gao 2009**	China	Hepatocellular carcinoma	Primary	52 (18-81)	204/36	60/180	70	42	39	83	57	49	0.029
**Jung 2016**	South Korea	Hepatocellular carcinoma	Primary	≤53: 44 1021 (28%) 267 (31%) >53: 41	69/16	23/62	43	19	17	90	69	59	<0.001
**Kan 2015**	China	Hepatocellular carcinoma	Primary	≤50: 56 1021 (28%) 267 (31%) >50: 72	108/20	105/23	30	5	0	50	15	10	0.001
**Umemoto 2015**	Japan	Hepatocellular carcinoma	Primary	64 ± 10	71/9	37/43	74	51	40	80	73	71	0.051
**Zeng 2011**	China	Hepatocellular carcinoma	Primary	53.1(35–68	109/32	31/32	38	-	-	85	-	-	0.000
**Gabrielson 2016**	USA	Hepatocellular carcinoma	Primary	61 (30–86)	50/15	30/35	85	85	-	53	45	-	0.029
**Wu 2009**	China	Hepatocellular carcinoma	Primary	48, 23–75	65/6	35/36	81	54	40	97	83	71	0.014
**Azuma 2014**	Japan	Lung cancer	Primary	66 (39-82)	91/73	82/164	-	-	38	-	-	56	0.039
**Chen 2012**	China	Lung cancer	Primary	≤54: 23 1021 (28%) 267 (31%) >54: 17	26/14	69/120	71	11	-	85	48	-	<0.001
**Cooper 2015**	USA	Lung cancer	Primary	-	477/201	628/678	95	73	62	84	54	44	0.023
**Jiang 2015**	China	Lung cancer	Primary	≤60: 15 1021 (28%) 267 (31%) >60: 64	39/40	50/79	100	91	84	83	74	70	0.042
**Kim 2015**	South Korea	Lung cancer	Primary	65 (45–81)	33/8	89/331	65	38	27	78	49	49	0.570
**Mu 2011**	China	Lung cancer	Primary	-	-	58/109	87	20	-	95	38	-	<0.005
**Velcheti 2014**	USA	Lung cancer	Primary	≤70: 232 1021 (28%) 267 (31%) >70: 80	260/37	56/155	78	43	27	87	61	51	0.028
**Yang 2014**	Taiwan	Lung cancer	Primary	≤70: 132 1021 (28%) 267 (31%) >70: 31	54/109	65/163	98	93	91	98	87	83	0.027
**Zhang 2014**	China	Lung cancer	Primary	≤58: 73 1021 (28%) 267 (31%) >58: 70	84/59	70/143	84	71	53	97	89	77	0.002
**Song 2016**	China	Lung cancer	Primary	<60: 207 ≥60: 178	198/187	186/199	99	71	40	99	79	52	0.069
**Inamura 2016**	Japan	Lung cancer	Primary	<60: 96 ≥60: 172	142/126	43/225	85	69	55	95	81	71	0.019
**Chen 2009**	China	Pancreatic cancer	Primary	<60: 61 ≥60: 55	76/23	18/40	32	8	-	84	58	17	0.001
**Nomi 2007**	Japan	Pancreatic cancer	Primary	-	-	20/51	48	12	-	78	24	-	0.016
**Wang 2010**	China	Pancreatic cancer	Primary	-	40/10	23/40	87	8	-	100	33	-	<0.001
**Gadiot 2011**	Netherlands	Merkel cell carcinoma	Primary	-	36/27	16/63	-	51	37	-	68	52	0.200
**Hino 2010**	Japan	Merkel cell carcinoma	Primary	68.84 ± 2.85	38/21	34/59	-	-	52	-	-	81	0.040
**Taube 2012**	USA	Merkel cell carcinoma	Primary	-	76/74	57/150	-	-	84	-	-	61	0.330
**Boorjian 2008**	USA	Urinary tract epithelial cell carcinoma	Primary	-	259/59	39/314	58	51	43	91	82	67	0.005
**Nakanishi 2006**	Japan	Urinary tract epithelial cell carcinoma	Primary	-	47/18	46/65	86	68	57	100	100	100	0.021
**Wang 2009**	China	Urinary tract epithelial cell carcinoma	Primary	-	31/5	36/50	91	68	-	100	100	-	0.020
**Xylinas 2014**	USA	Urinary tract epithelial cell carcinoma	Primary	65.9 (60.5e72.2)	244/58	76/226	83	66	63	95	82	69	0.020
**Kim 2016**	South Korea	Oral squamous cell cancer	Primary	65 (45–81)	33/8	90/43	97	83	80	98	83	75	0.625
**Lin 2015**	Taiwan	Oral squamous cell cancer	Primary	<56: 162 ≥56: 143	236/69	133/172	81	62	56	81	62	58	0.225
**Cho 2011**	South Korea	Oral squamous cell cancer	Primary	<59: 20 ≥59: 25	32/13	26/45	72	51	43	72	63	63	0.012
**Oliveira 2015**	USA	Oral squamous cell cancer	Primary	<60: 62 ≥60: 34	85/11	47/96	81	47	-	61	18	-	0.044
**Ukpo 2013**	USA	Oral squamous cell cancer	Primary	55.8 ± 9.4	186/23	84/181	89	74	62	97	76	64	0.730

### PD-L1 expression and OS across all studies

**Figure 2:**
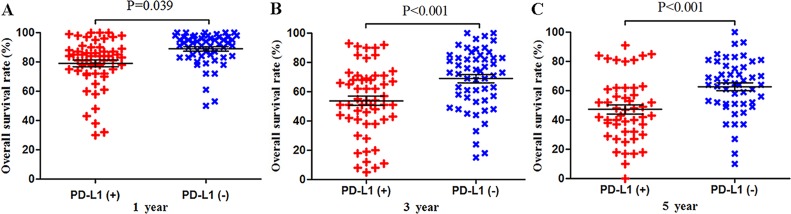
Scatter plot of OS at 1, 3 and 5 years for patients positive or negative for PD-L1 expression Data come from the entire patient population.

Meta-analysis of data from all 59 studies showed that the median OS rate was significantly lower in PD-L1-positive patients than in PD-L1-negative patients at 1 year (P = 0.039), 3 years (P < 0.001) and 5 years (P < 0.001; Figure 2). The RR for OS at the three time points (and associated 95% confidence intervals [CIs]) were 2.02 (1.56-2.60), 1.57 (1.34-1.83) and 1.43 (1.24-1.64) (Table 2 and Figure 2).

**Table 2:** Meta-analysis of possible associations between PD-L1 expression and overall survival in patients with solid tumors

Group or subgroup	N	PD-L1(+/-)	1 year OS			3 year OS			5 year OS		
			RR (95 % CI)	*P*	*I^2^*	RR (95 % CI)	*P*	*I^2^*	RR (95 % CI)	*P*	*I^2^*
**All studies**	59	6028/13976	2.02 (1.56-2.60)	<0.001	84	1.57 (1.34-1.83)	<0.001	91	1.43 (1.24-1.64)	<0.001	92
**Ethnic subgroups**											
Asian	35	2211/4126	1.83 (1.61-2.08)^*^	<0.001	49	1.57 (1.39-1.77)	<0.001	74	1.44 (1.31-1.58)	<0.001	92
Non-Asian	24	3817/9850	1.98 (1.27-3.09)	0.003	90	1.60 (1.18-2.17)	0.003	95	1.39 (1.08-1.78)	0.009	95
**Tumor origin**											
Gastrointestinal tumors	24	1778/3206	2.12 (1.45-3.09)	<0.001	86	1.52 (1.23-1.89)	<0.001	91	1.40 (1.17-1.67)	<0.001	91
Other tumors	35	4250/10770	1.79 (1.33-2.40)	<0.001	86	1.61 (1.30-1.98)	<0.001	92	1.47 (1.23-1.75)	<0.001	91
**Tumor type**											
Breast cancer	5	1647/5677	1.80 (0.60-5.42)	0.30	79	1.79 (0.77-4.19)	<0.18	95	1.80 (0.68-4.73)	<0.24	96
Esophageal cancer	4	187/252	1.90 (0.69-5.21)	0.21	70	2.77 (1.78-4.30)^*^	<0.001	48	3.55 (2.63-5.65)^*^	<0.001	0
Gastric carcinoma	7	421/875	2.48 (1.80-3.41)^*^	<0.001	18	1.63 (1.43-1.87)^*^	<0.001	32	1.45 (1.18-1.79)	<0.001	79
Hepatocellular carcinoma	7	321/339	1.87 (1.01-3.46)	0.04	78	1.40 (0.92-2.15)	0.12	84	1.58 (1.11-2.25)	0.01	83
Lung cancer	11	1396/2366	1.39 (0.69-2.81)	0.36	88	1.17 (0.84-1.63)	0.35	92	1.16 (0.86-1.57)	0.32	93
Pancreatic cancer	3	61/131	3.43 (2.06-5.73)^*^	<0.001	15	1.48 (1.06-2.06)^*^	0.02	0	-	-	-
Merkel cell carcinoma	3	107/272	-	-	-	-	-	-	1.01 (0.41-2.99)	0.85	89
urinary tract epithelial cell carcinoma	4	197/655	6.24 (3.62-10.74)^*^	<0.001	0	3.43 (1.50-7.84)	0.003	75	1.79 (0.86-3.70)	0.12	82
Oral squamous cell cancer	5	380/537	1.05 (0.58-1.93)	0.87	63	0.95 (0.72-1.26)	0.72	55	1.07 (0.89-1.29)^*^	0.45	0
Renal cell carcinoma	7	208/572	3.38 (2.13-5.39)^*^	<0.001	24	4.14 (2.07-8.26)	<0.001	81	2.57 (1.46-4.52)	<0.001	79
Colorectal cancer	3	788/1609	1.17 (0.27-5.06)	0.84	95	0.94 (0.33-2. 67)	0.90	96	1.16 (0.55-2.45)	0.69	95

N, number of studies; OS, overall survival; RR, risk ratio; 95% CI, 95% confidence interval

^*^ These meta-analyses were performed using a fixed-effects model. All other meta-analyses were performed using a random-effects model.

### Subgroup analysis by tumor type

Given the significant heterogeneity in the meta-analysis involving all 59 studies, we performed a series of subgroup analyses to eamine the possible correlation between PD-L1 expression and OS. PD-L1 expression was associated with worse 1-year OS for the following types of solid tumor (Table 2): gastric cancer, 2.48 (1.80-3.41); renal cell carcinoma, 3.38 (2.13-5.39); and hepatocellular carcinoma, 1.87 (1.01-3.46). PD-L1 expression was associated with worse 3-year OS for the following cancers: esophageal cancer, 2.77 (1.78-4.30); gastric cancer, 1.63 (1.43-1.87); pancreatic cancer, 1.48 (1.06-2.06); and renal cell carcinoma, 4.14 (2.07-8.26). PD-L1 expression was associated with worse 5-year OS for esophageal cancer, 3.55 (2.63-5.65); gastric cancer, 1.45 (1.18-1.79); hepatocellular carcinoma, 1.58 (1.11-2.25); and renal cell carcinoma, 2.57 (1.46-4.52).

**Figure 3:**
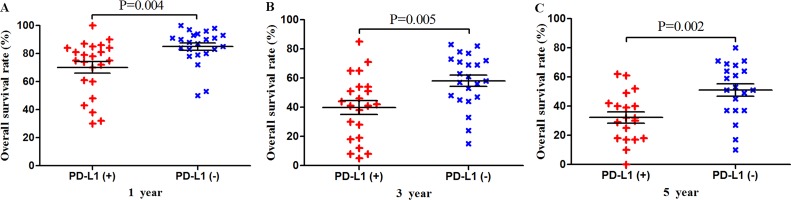
Scatter plot of OS at 1, 3 and 5 years for patients positive or negative for PD-L1 expression Data come from the subset of patients with gastrointestinal tumors.

Among the subset of 4,984 patients with gastrointestinal tumors, 1,778 (35.6%) were PD-L1-positive and 3,206 (64.4%) were PD-L1-negative. PD-L1 expression was associated with significantly worse OS at 1 year (P = 0.004), 3 years (P = 0.005), and 5 years (P = 0.002; Figures 3 and 7). The corresponding RRs and 95% CIs were 2.12(1.45-3.09), 1.52 (1.23-1.89), and 1.40 (1.17-1.67) (Table 2).

**Figure 4:**
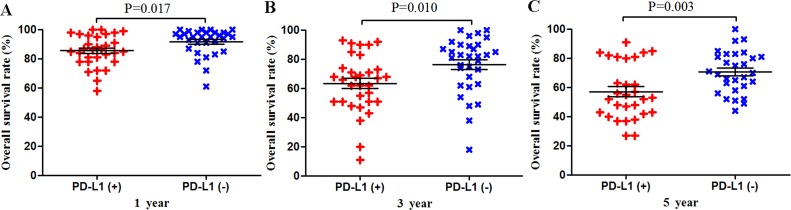
Scatter plot of OS at 1, 3 and 5 years for patients positive or negative for PD-L1 expression Data come from the subset of patients with non-gastrointestinal tumors.

**Figure 5:**
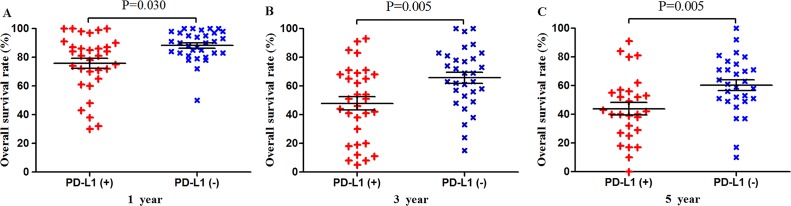
Scatter plot of OS at 1, 3 and 5 years for patients positive or negative for PD-L1 expression Data come from the subset of Asian patients.

**Figure 6:**
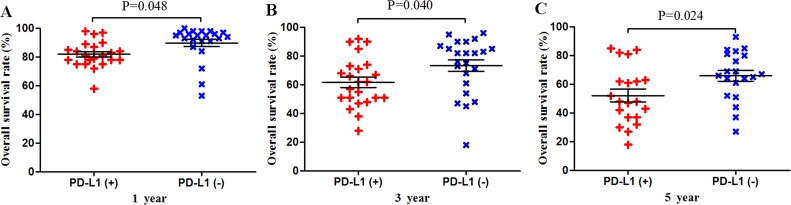
Scatter plot of OS at 1, 3 and 5 years for patients positive or negative for PD-L1 expression Data come from the subset of non-Asian patients.

**Figure 7:**
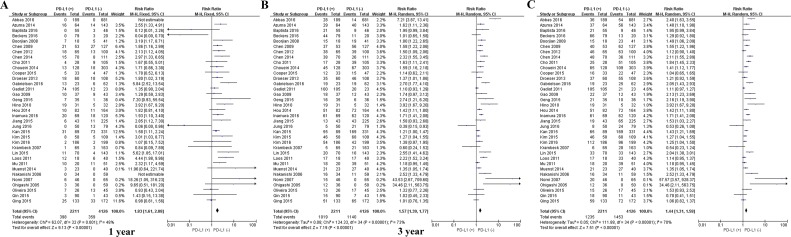
Forrest plot of OS at 1, 3 and 5 years for patients positive or negative for PD-L1 expression Data come from the subset of patients with gastrointestinal tumors.

**Figure 8:**
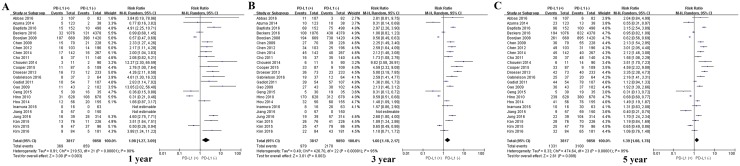
Forrest plot of OS at 1, 3 and 5 years for patients positive or negative for PD-L1 expression Data come from the subset of patients with non-gastrointestinal tumors.

Among the subset of 4,309 patients with non-gastrointestinal tumors, 2,298 (53.3%) were PD-L1-positive and 1,404 (59.3%) were PD-L1-negative. PD-L1 expression was associated with significantly worse OS at 1 year (P = 0.017), 3 years (P = 0.010) and 5 years (P = 0.003; Figures 4 and 8). The corresponding RRs and 95% CIs were 1.79 (1.33-2.40), 1.61 (1.30-1.98), and 1.47 (1.23-1.75) (Table 2).

### Subgroup analysis by patient ethnicity

**Figure 9:**
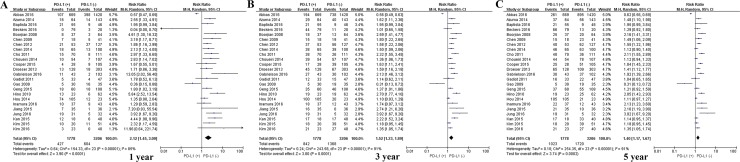
Forrest plot of OS at 1, 3 and 5 years for patients positive or negative for PD-L1 expression Data come from the subset of Asian patients.

Among the subset of 6,337 Asian patients, 2,211 were PD-L1-positive and 4,126 were PD-L1-negative. PD-L1 expression was associated with significantly lower OS at 1 year (P = 0.030), 3 years (P = 0.005) and 5 years (P = 0.005; Figures 5 and 9). The corresponding RRs and 95% CIs were 1.86 (1.61-2.08), 1.57 (1.39-1.77), and 1.44 (1.31-1.58) (Table 2).

**Figure 10:**
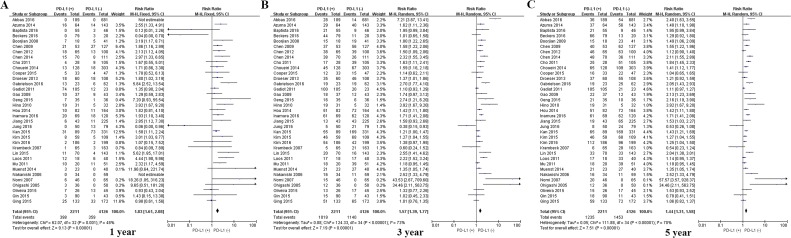
Forrest plot of OS at 1, 3 and 5 years for patients positive or negative for PD-L1 expression Data come from the subset of non-Asian patients.

Among the subset of 13,667 non-Asian patients, 3,817 were PD-L1-positive and 9,850 were PD-L1-negative. PD-L1 expression was associated with significantly lower OS at 1 year (P = 0.048), 3 years (P = 0.040) and 5 years (P = 0.024; Figures 6 and 10). The corresponding RRs and 95% CIs were 1.98 (1.27-3.09), 1.60 (1.18-2.17), and 1.39 (1.08-1.78) (Table 2).

## DISCUSSION

While studies published more than a decade ago established that PD-L1 promotes cancer immune escape [82, 83] and that blocking PD-L1 can improve the anti-tumor efficacy of anti-tumor responses [84–86], whether PD-L1 expression by solid tumors negatively affects patient prognosis remains unclear. Here we reviewed 59 studies involving 20,004 patients with 11 types of solid tumors and found strong evidence that PD-L1 expression is associated with significantly lower OS at 1, 3 and 5 years. This effect was observed in meta-analyses involving all patients as well as several subgroups of patients stratified by ethnicity and tumor type.

PD-L1 positive expression is associated with viral infection and chronic inflammation [87]. Expression of PD-L1 and/or PD-1 has been described for numerous types of cancers associated with viral infection [88], including polycyclic virus-associated Merkel cell carcinoma [89], hepatitis B virus-associated hepatocellular carcinoma [33], human papillomavirus-associated head and neck cancer, and Epstein-Barr virus-related nasopharyngeal carcinoma [90]. In patients with hepatocellular carcinoma, PD-L1 expression was significantly higher in tumor macrophages than in matched normal tissues, and expression correlated with tumor grade [25].

Our results are consistent with previous reports that PD-L1 expression is associated with worse 5-year outcome in patients with gastrointestinal carcinomas such as esophageal cancer and gastric cancer [70, 79] as well as colorectal cancer [25]. The precise mechanisms whereby PD-L1 expression may worsen prognosis are unknown; When PD-1 binds to the ligand PD-L1 (B7-H1) expressed on the surface of tumors, PD-1 has been shown to promote tumor cell-specific T cell inactivation or apoptosis [12].

The results of this meta-analysis should be interpreted cautiously because of several limitations. One is the lack of a standardized assay and cut-off value for classifying patients as PD-L1-positive. This may help explain the high heterogeneity observed across the included studies. Another limitation is our exclusion of gray literature, which may have increased the risk of publication bias and selection bias.

Despite these limitations, this large meta-analysis provides strong evidence that expression of PD-L1 may be a meaningful index for predicting prognosis in a wide variety of patients with solid tumors. These findings justify more focused prognostic studies in well-defined patient populations in which a panel of clinically relevant outcomes beyond only OS are considered.

## MATERIALS AND METHODS

### Literature search

PubMed, EMBASE, Web of Science and EBSCO were searched through 15 January 2017 to identify cohort and case-control studies examining the relationship between PD-L1 expression and prognosis of patients with solid tumors. The following search terms were used: *programmed death-ligand 1, PD-L1, B7-H1, CD274* and *solid tumor*.

### Inclusion and exclusion criteria

To be included in our meta-analysis, studies had to involve (1) primary solid tumors in human patients; (2) The main content of the articles is to analyze the relationship between the expression of PD-L1 and the prognosis of solid tumors in patients; (3) a hospital-based or population-based case-control or cohort design, regardless of sample size; (4) immunohistochemical assay of PD-L1 expression as high and low PD-L1 expression; (5) all patients underwent surgery; and (6) adequate reporting of overall survival (OS) data. When eligible studies involved overlapping patient populations, only the most recent or complete report was included. Studies were excluded if they were letter, summary of meeting and review; if they were published in a language other than English; or if they failed to report adequate data; or they investigated metastatic tumors. Gray literature (Reports and papers that were not published in PubMed, EMBASE, Web of Science and EBSCO) was not included into this study. Reference lists within identified articles were also searched manually to identify additional articles.

### Meta-analysis outcomes

The primary outcome in the meta-analysis was OS. This outcome was compared between patients showing high or positive PD-L1 expression and patients showing low or no expression, as defined within the individual studies.

### Data collection

Two researchers (P.-C.Y, X.X) independently screened studies for inclusion. Disagreements were resolved by discussion and, when necessary, consultation with a third author (S.Z). The first author's name, year of publication, country, number of patients, and tumor type were extracted from each study, and OS results for 1, 3 and 5 years were extracted from tables or Kaplan-Meier curves.

### Statistical analysis

Forest plots of OS were generated using RevMan 5.3 (Cochrane Collaboration, Copenhagen, Denmark). Weighted risk ratio (RR) estimates were generated from pooled data using Mantel-Haenszel random-effects meta-analysis, unless no statistically heterogeneity, in which case fixed-effects meta-analysis was performed. Statistical heterogeneity in meta-analyses was assessed using Cochrane's Q and I^2^statistics. Survival results were analyzed using scatter plots generated in Prism 5 (Graphpad Software, San Diego, USA). The results for different patient groups were compared using the log-rank test. The threshold of statistical significance was defined as P < 0.05.
